# Precision Grip in Congenital and Acquired Hemiparesis: Similarities in Impairments and Implications for Neurorehabilitation

**DOI:** 10.3389/fnhum.2014.00459

**Published:** 2014-06-30

**Authors:** Yannick Bleyenheuft, Andrew M. Gordon

**Affiliations:** ^1^Institute of Neuroscience, Université Catholique de Louvain, Brussels, Belgium; ^2^Department of Biobehavioral Sciences, Teachers College, Columbia University, New York, NY, USA

**Keywords:** fingertip force, grip force, precision grip, cerebral palsy, stroke

## Abstract

**Background:** Patients with congenital and acquired hemiparesis incur long-term functional deficits, among which the loss of prehension that may impact their functional independence. Identifying, understanding, and comparing the underlying mechanisms of prehension impairments represent an opportunity to better adapt neurorehabilitation.

**Objective:** The present review aims to provide a better understanding of precision grip deficits in congenital and acquired hemiparesis and to determine whether the severity and type of fine motor control impairments depend on whether or not the lesions are congenital or acquired in adulthood.

**Methods:** Using combinations of the following key words: fingertip force, grip force, precision grip, cerebral palsy, stroke, PubMed, and Scopus databases were used to search studies from 1984 to 2013.

**Results:** Individuals with both congenital and acquired hemiparesis were able to some extent to use anticipatory motor control in precision grip tasks, even if this control was impaired in the paretic hand. In both congenital and acquired hemiparesis, the ability to plan efficient anticipatory motor control when the less-affected hand is used provides a possibility to remediate impairments in anticipatory motor control of the paretic hand.

**Conclusion:** Surprisingly, we observed very few differences between the results of studies in children with congenital hemiplegia and stroke patients. We suggest that the underlying specific strategies of neurorehabilitation developed for each one could benefit the other.

## Introduction

Brain lesion is the most prevalent cause of physical disability: cerebral palsy occurs in 1 out of 303 live births (Murphy et al., 1993; Stanley et al., 2000; Center for Disease Control and Prevention, 2014a), and stroke occurs in 0.7% of adults below 45 years, 3% of adults 45–64 years old, and in 8.3% of adults older than 65 years (Center for Disease Control and Prevention, 2014b). Manual dexterity is frequently impaired in these patients, resulting in long-term functional deficits. Thus, it is important to identify the underlying causes of these deficits in order to better focus neurorehabilitation based on concrete scientific evidence, with the aim of improving patients’ function during typical daily activities.

Various levels of sensorimotor processing can be affected by unilateral brain lesions and influence dexterity. During the last 25 years, precision grip tasks, allowing the measure of fingertip forces during manipulation, have been developed to study skilled manual dexterity (Johansson and Westling, 1984, 1988a; Johansson, 2002). This approach has also been used to delineate the mechanisms underlying prehensile control in children with hemiplegic cerebral palsy (HCP) and adults with hemiplegic stroke. The present review provides an overview of precision grip impairments in these patients, with the aim of comparing impaired mechanisms in congenital versus adult occurrence of the lesion. Surprisingly, despite many congruent disabilities in these pathologies, we found only a few papers in the literature including both children with HCP and adult stroke patients: one describing the effects of upper-extremity casting (Lanin et al., 2007) and one on oral anti-spasticity medication (Montané et al., 2004). The similarities and differences in the mechanisms underlying precision grip in both pathologies have not been examined. Due to differences in the pathophysiology after lesion, differential impairments are generally expected. In children with HCP, depending on the age the CNS damage occurs *in utero*, there may be a complete reorganization of the corticospinal projections, whereby movements of the paretic hand may be controlled entirely by the contralesional hemisphere (Staudt et al., 2004; Eyre et al., 2007; Gordon et al., 2013). Such structural plasticity does not occur following CNS injury later in life (Newton et al., 2006). Despite the great disparity in age (generally 6–14 years in children and more than 60 years in adults) and the variation in lesion locations, we document a remarkable similarity in fingertip force impairments, highlighting impairments in timing, coupling, and amplitude of prehensile forces. The specific impairments documented may allow us to more precisely focus neurorehabilitation.

### Predictive and reactive grip force control

**Figure 1:**
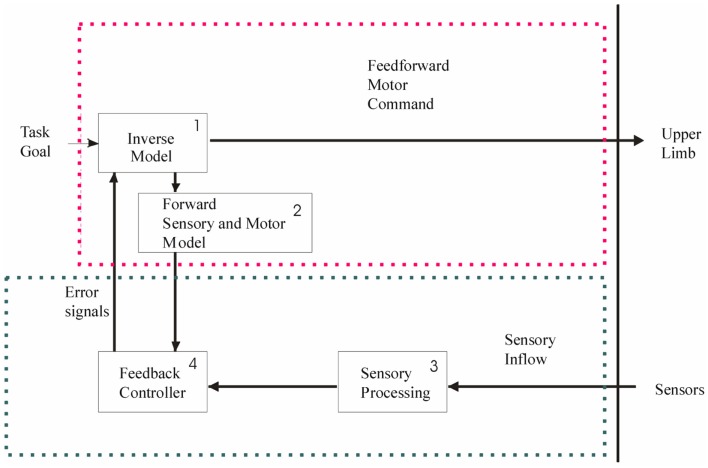
**To achieve a precision grip movement, the goal of the task is sent to an inverse model (1) that generates a motor command**. Due to this motor command, a movement of the upper limb is generated. In parallel, a forward sensory and motor model (2) is generated. This forward model predicts the movement induced by the motor command and estimates the sensory feedback of the new state of the hand and arm. It allows comparison with actual feedback (4) and consequently there is an updating of the motor command. Actual feedback emanates from sensors and is transmitted to the feedback controller (4) after sensory processing (3). The red dotted frame represents the feedforward components, and the green frame denotes the feedback components. Both can be affected at different levels in unilateral brain lesions, with consequential impairment to precision grip.

Two types of control mechanisms are used to perform skilled hand movements: predictive mechanisms – that enable one to anticipate the movement on the basis of sensorimotor memories of the manipulated object, and reactive mechanisms – that allow for correction of the movements/forces. Reactive mechanisms are typically feedback loops emanating from sensory and/or visual afferents. It is generally accepted that predictive mechanisms, also called feedforward or anticipatory mechanisms, are based on internal models in the central nervous system (Wolpert and Ghahramani, 2000). These include: (1) an inverse model that uses the current state of the limb and the specific context to generate an appropriate motor command, (2) a forward sensory and motor model that predicts the movement resulting from the motor command and estimates the sensory feedback of the new state of the limb, allowing a comparison (3) with actual feedback (4) and subsequent adjustment of the motor command (Figure 1). It is the close interaction between predictive and reactive mechanisms that allows for the production of smooth movements.

**Figure 2:**
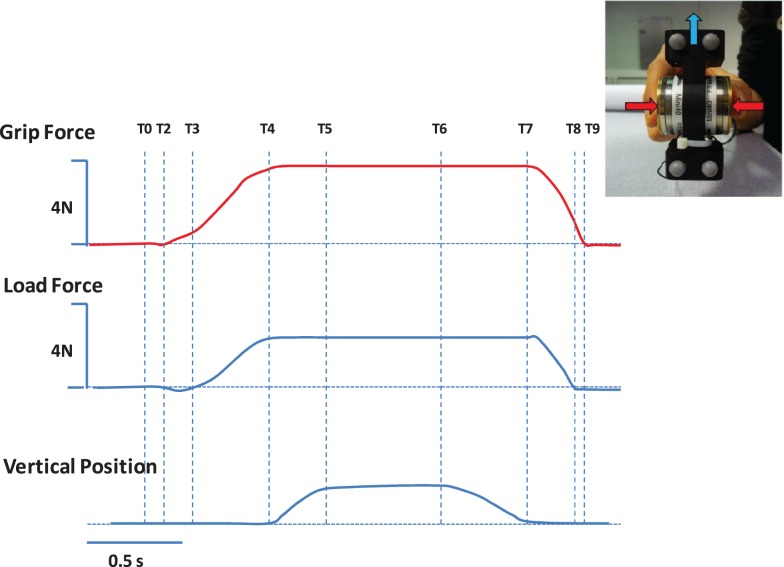
**Representation of the grip (red) and load (blue) forces applied on a handheld object during a grip–lift task, as well as the vertical position (lower panel) of the handheld object**. The different phases of the grip–lift task are highlighted with dotted lines. T0–T2, the contact between fingers and the object is initiated in a quick succession. T2–T3, preload phase, GF increases prior to LF onset. T3–T4, loading phase, GF and LF subsequently increase in parallel. T5–T6, static phase, followed by the release of the object including a replacement phase (T6–T7) of a subsequent rapid decrease in the grip and load forces (T7–T8) until the thumb and index fingers are released from the object (T8–T9).

In the context of brain lesions, prehension deficits could be explained by impairments in predictive and/or reactive mechanisms. This is of particular interest for the design of rehabilitation programs. These mechanisms have been well-studied and described in healthy adults and typically developing children, especially through analysis of the timing and magnitude of grip force (GF) and the tangential load forces (LF) during grip–lift manipulation (Johansson and Westling, 1984, 1988a; Forssberg et al., 1991, 1992, 1995; Gordon et al., 1992; Witney et al., 2004). In healthy adults, there is a well-described sequence of events comprising a typical coordination pattern (Johansson and Westling, 1984, 1988a) during a grip–lift task (see Figure 2): first the contact between fingers and the object is initiated in a quick succession (T0–T2). During the preload phase (T2–T3), GF increases prior to LF onset. GF and LF subsequently increase in parallel during the loading phase (T3–T4). Forces rates are characterized by single peaks that are well-timed. After the parallel increase of forces, the forces are steadily maintained during a static phase (T5–T6) in which the object is held in the air, that is followed by the release of the object including a replacement phase (T6–T7) an a subsequent rapid decrease in the grip and LF (T7–T8) until the thumb and index fingers are released from the object (T8–T9).

The adaptation of the forces to the object’s properties during precision grip tasks requires the use of tactile (Westling and Johansson, 1987) and weight-related information (Johansson, 1996). Adaptation to various load and frictional conditions has been well-established. However, such information about the object’s physical properties is not instantly available due to delays in the transmission of sensory information. Therefore, to manage objects with adequate forces in everyday life, GF and LF forces are planned in an anticipatory manner on the basis of internal representations of the objects from prior manipulatory experience (Johansson and Westling, 1987, 1988a; Gordon et al., 1993), i.e., before initiating the movement.

In typically developing children, this anticipatory coordination of GF and LF matures until approximating that of adults at the age of 6–8 years (Forssberg et al., 1991). Before that age, the forces do not increase in parallel, and there are negative LFs at the start and excessive and multiple increments in GF during the loading phase (Forssberg et al., 1991).

This immature strategy has been interpreted as a control strategy relying mainly on feedback. Due to the delay of the feedback, the adjustment of the motor command is sequential, which does not allow smooth movements. With continuing maturation and experience during childhood, children utilize internal representations of the objects, allowing a predictive (anticipatory) control of the movement (Forssberg et al., 1991).

### Predictive and reactive GF control in a bimanual context

The integration of the precise GF and LF coupling in a bimanual context have been studied recently when opposite or concomitant patterns of force are required in the hands: a handheld object had to be placed on the top of another object statically held in the other hand (Islam et al., 2011) or two pieces of a handheld object had to be pulled apart (Smits-Engelsman et al., 2011). These tasks, when performed with healthy adults, demonstrated coordinated actions of both hands.

The use of one hand to generate a rapid increase of LF to an object held by the opposite hand has also been used as a model of coordination. In that context, a predictive increase in GF is observed before the rapid LF increase (Eliasson et al., 1995; Bleyenheuft et al., 2009). After the brisk LF increase, there is a systematic second increase in GF, programed as a predictive action that allows an optimal stabilization of the object around the brisk force increase. Self-induced brisk load increases also mature during childhood, approaching adult values at 9–10 years of age (Eliasson et al., 1995; Bleyenheuft and Thonnard, 2010a).

## Methods

### Data sources and literature selection

PubMed and Scopus electronic databases were searched using combinations of the following terms: GF, fingertip force, and precision grip either with “cerebral palsy” or “stroke.” Studies from 1984 to 2013 were retained. An additional hand-search was conducted in the reference lists of the articles meeting the search criteria. The search procedure included only studies in English. Studies focused on heat and pain, as well as animal studies and studies dedicated to other clinical forms of CP (e.g., diplegic or bilateral CP) were excluded.

## Results

**Figure 3:**
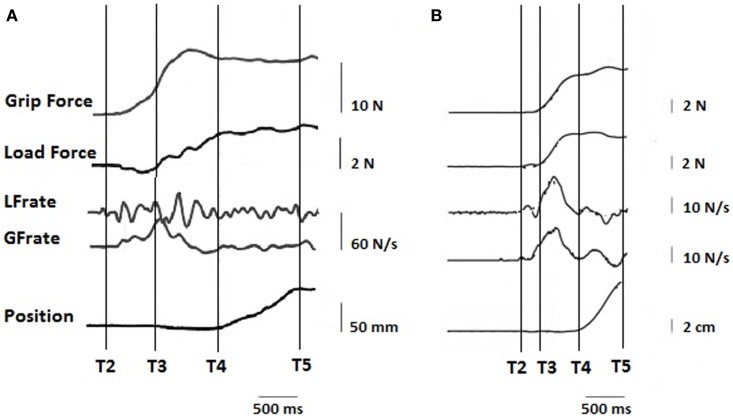
**Representation of typical traces on the paretic hand of (A) a child with congenital hemiparesis and (B) a stroke patient**. T2–T3, preload phase, T3–T4, loading phase, T5 start of static phase.

Skilled hand movements both in pediatric HCP and adult stroke patients are impaired (Eliasson et al., 1991, 1992, 1995a, 2006; Steenbergen et al., 1998, 2008; Forssberg et al., 1999; Gordon and Duff, 1999; Gordon et al., 1999, 2003, 2006; Eliasson and Gordon, 2000; Duff and Gordon, 2003; Duque et al., 2003; Hermsdörfer et al., 2003; Nowak et al., 2003; Takahashi and Reinkensmeyer, 2003; Smits-Engelsman et al., 2004; Wenzelburger et al., 2005; Blennerhassett et al., 2006, 2008; McDonnell et al., 2006; Mutsaarts et al., 2006; Raghavan et al., 2006; Mackenzie et al., 2009; Quaney et al., 2010; Seo et al., 2010; van Elk et al., 2010; Naik et al., 2011; Prabhu et al., 2011; Bleyenheuft and Gordon, 2013). Table 1 highlights the precision grip impairments that have been observed on the more affected (paretic) hand of children with HCP and adults with stroke. Figure 3 provides examples of forces coordination in the more affected hand of a child with congenital hemiplegia (A) and a stroke patient (B).

**Table 1:** **Deficits of precision grip in children with HCP and stroke patients**.

	HCP	Stroke
**a. Preload phase**
Push down object before lifting	✓Eliasson et al. (1991); Duque et al. (2003)	✓McDonnell et al. (2006)
Longer duration	✓Eliasson et al. (1991); Duque et al. (2003)	✓Hermsdörfer et al. (2003); Nowak et al. (2003); Takahashi and Reinkensmeyer (2003); Wenzelburger et al. (2005); Blennerhassett et al. (2006); McDonnell et al. (2006); Raghavan et al. (2006); Quaney et al. (2010); Naik et al. (2011)
**b. Loading phase**
Asynchronous onset of GF and LF	✓Eliasson et al. (1991, 1992, 1995a, 2006); Forssberg et al. (1999); Gordon and Duff (1999); Gordon et al. (1999); Duque et al. (2003)	✓Blennerhassett et al. (2006); McDonnell et al. (2006)
Excessive GF at LF increase	✓Eliasson et al. (1991, 1992); Duque et al. (2003)	✓Hermsdörfer et al. (2003); Wenzelburger et al., 2005; Blennerhassett et al. (2006); McDonnell et al. (2006)
Multiple increments in force rates	✓Eliasson et al. (1991, 1992); Duque et al. (2003)	✓Blennerhassett et al. (2006); McDonnell et al. (2006)
**c. GF**
Higher	✓Eliasson et al. (1991, 1992, 1995a); Forssberg et al. (1999); Gordon and Duff (1999); Gordon et al., 1999; Duque et al. (2003)	✓Hermsdörfer et al. (2003); Nowak et al. (2003); Wenzelburger et al. (2005); Blennerhassett et al. (2006); McDonnell et al. (2006)
Altered digit direction		✓Seo et al. (2010)
**d. Release**
Sequential force coordination	✓Duff and Gordon (2003); Gordon et al. (2003)	✓Naik et al. (2011)
**e. Adaptation to different weights**
Need more trials to adapt	✓Eliasson et al. (1992); Steenbergen et al. (1998); Gordon and Duff (1999)	✓McDonnell et al. (2006); Raghavan et al. (2006)
**f. Adaptation to different frictions**
Need more trials to adapt	✓Eliasson et al. (1995a); Duff and Gordon (2003)	
**g. Predictive abilities**
Demonstrated	✓Gordon et al. (1999); Duff and Gordon (2003); Gordon et al. (2006); Steenbergen et al. (2008)	✓Hermsdörfer et al. (2003); Nowak et al. (2003); Hermsdörfer et al. (2004)
Anticipation perturbed	✓Eliasson et al. (1992); Gordon et al. (2006); Mutsaarts et al. (2006); Steenbergen and Gordon (2006); Bleyenheuft and Thonnard (2010a)	✓Nowak et al. (2003); Takahashi and Reinkensmeyer (2003); Hermsdörfer et al. (2004); McDonnell et al. (2006); Blennerhassett et al. (2007)
Transfer from non-paretic to paretic	✓Gordon et al. (1999); Steenbergen et al. (2008)	✓Raghavan et al. (2006)

### Children with HCP

In children with HCP, the fingertip forces deviate from that observed in age-matched typically developing children: the timing of GF and LF onset is disrupted, GF is excessive, especially at the onset of LF increase (Eliasson et al., 1991, 1992, 1995a; Forssberg et al., 1999; Gordon and Duff, 1999; Gordon et al., 1999; Hermsdörfer et al., 2003) and present multiple successive increments that can also be observed in the force rates. The object is frequently pushed down toward the support, generating a negative LF before initiating the lifting phase. While encountering these impairments in precision grip, children with HCP demonstrated some ability to adapt to various weight and friction conditions, provided they had a greater number of trials to adapt to new conditions (Steenbergen et al., 1998; Gordon and Duff, 1999). As shown in a longitudinal study, these impairments are not increasing with time (Eliasson et al., 2006), which is congruent with other studies on deficits in maximal grip strength and isometric finger forces (Smits-Engelsman et al., 2004). During the release of a handheld object, children with HCP demonstrate also a sequential coordination of forces (Eliasson and Gordon, 2000). However these forces are adapted to the weight of the object (Eliasson and Gordon, 2000). Impairments in replacement and release of handheld objects are increased when speed and/or accuracy are imposed (Gordon et al., 2003). However, it is not clear if the impairments observed are linked to predictive or reactive control of precision grip.

Predictive abilities in grasp control were demonstrated in children with cerebral palsy, since they are able to form and retain internal representations of new objects (Duff and Gordon, 2003); there is an influence of bimanual tasks on the performance of the more and less-affected hand (Steenbergen et al., 2008) and these children have the ability to transfer learned forces from the less-affected to the more affected hand (Gordon et al., 1999, 2006). However these predictive abilities (Mutsaarts et al., 2006; Steenbergen and Gordon, 2006; Bleyenheuft and Thonnard, 2010b), as well as their motor imagery (van Elk et al., 2010), are clearly impaired. It has been suggested that the impaired precision grip of children with HCP is the consequence of an inability to use internal models of manipulated objects. This could be due to inefficient feedback from the paretic hand (Gordon and Duff, 1999; Gordon et al., 1999) or to high-level deficits in sensorimotor integration (Eliasson et al., 1992; Bleyenheuft and Thonnard, 2010b). The role of predictive and/or reactive deficits in the precision grip of children with HCP has been further studied using a paradigm (self-or-examiner-imposed load to a handheld object) where both were tested independently (Bleyenheuft and Thonnard, 2010b). Predictive and reactive conditions were observed in the paretic hand: in the paretic hand, children were able to anticipate events prior to the brisk load increase, but were unable to pursue predictive control afterward (Bleyenheuft and Thonnard, 2010b). Interestingly, these predictive motor control deficits were not observed in the less-affected hand. The less-affected hand had been previously studied in paradigms showing that while anticipatory control is present (Gordon et al., 1999, 2006; Steenbergen et al., 2008), subtle deficits can be observed (Steenbergen et al., 1998; Forssberg et al., 1999; Gordon et al., 1999; Mutsaarts et al., 2006; van Elk et al., 2010). These subtle deficits are congruent with those observed on the more affected hand but are not as pronounced: compared to controls, manipulation with the less-affected hand demonstrates lower force rates, increased duration of preload phase, negative LF during the preload phase, overall higher GF, and an increase of forces that is less parallel (Gordon et al., 1999). Nevertheless, anticipatory control is present (Gordon et al., 1999, 2006; Steenbergen et al., 2008) suggesting lateralized impairments in motor planning underlying precision grip in HCP, which could reflect a lateralized deficit in the sensorimotor integration (Prabhu et al., 2011). In bimanual tasks, however, children with HCP demonstrate less accurate performance in both hands when compared to unimanual use (Islam et al., 2011; Smits-Engelsman et al., 2011), suggesting that while dissociated in unimanual motor planning, each effector can be influenced by the other in a bimanual context.

These results, consistent with previous findings, suggest that the abilities of the less-affected side could be used in neurorehabilitation to improve motor control of the paretic side. For a recent systematic review on sensorimotor deficits in children with HCP, see also Bleyenheuft and Gordon (2013).

### Stroke patients

Precision grip in the paretic hand of stroke patients is also impaired. The GF is generally higher – which is also seen already at the onset of the LF increase – and prolongations are observed in the timing of the movement: there is an asynchronous onset of GF and LF, leading to a longer preload phase and discontinuous force increase (Hermsdörfer et al., 2003; Nowak et al., 2003; Takahashi and Reinkensmeyer, 2003; Wenzelburger et al., 2005; Blennerhassett et al., 2006, 2008; McDonnell et al., 2006; Raghavan et al., 2006; Quaney et al., 2010). This longer preload phase is also correlated to clinical measures of handgrip limitations (Blennerhassett et al., 2008). Similar to congenital hemiparesis, the stroke patients push the object on the table before lifting it (McDonnell et al., 2006). There is a reduced ability to adapt to changes in the object’s weight (McDonnell et al., 2006; Raghavan et al., 2006), although some adaptations still occur. The release phase is also impaired, showing sequential force coordination (Naik et al., 2011). Altered digit GF direction during prehension tasks in stroke patients has a direct impact on grip (Seo et al., 2010), but can be improved through visual force feedback (Seo et al., 2011). Deficits in prediction of the inertial load profile arising from voluntary movement with handheld objects were also demonstrated in patients with acute stroke performing lift-and-hold tasks and point-to-point movements (Nowak et al., 2003). Since the same type of impairments has been delineated in patients with cortical and subcortical stroke – increased GF and deficits in temporal coupling – the authors suggested that the internal models responsible for precise regulation of forces were impaired. While these models are thought to be formed in the cerebellum (Wolpert et al., 1998; Wolpert and Flanagan, 2001), Nowak et al. (2003) argued that cortical and subcortical structures should be involved in the subsequent process of issuing motor commands. Studies investigating the ability of patients with chronic stroke to learn anticipation (either by force disturbance during a reaching task (Wenzelburger et al., 2005), or presenting objects with different shapes (Raghavan et al., 2010) showed an impaired ability to adapt. However, a partial ability to form and use internal models was retained (Wenzelburger et al., 2005). In contrast, Hermsdörfer et al. (2003, 2004) suggested that internal models are preserved in patients with chronic cerebral stroke since the feedforward nature of the GF coupling with LF was preserved during object oscillations. The difference observed in these two paradigms may be linked to the fact that the GF–LF coordination was studied, respectively, in discrete and rhythmic coordination patterns. We hypothesize that rhythmic (more robust) coordination might be preserved while anticipatory motor control needed for discrete tasks might be impaired. These high-order motor planning deficits observed in patients with subcortical subacute and chronic stroke are ameliorated by transfer of information from the unaffected hemisphere (at least in right hemiparesis) (Raghavan et al., 2006).

The precision grip impairments described for various stroke patients are of importance because there is an impact of unimanual dysfunction on bimanual tasks, and therefore on activities of daily living (Müller and Dichgans, 1994). Excessive GF and deficits in the temporal coupling of the forces have been observed in the paretic hand of stroke patients (cortical, subcortical, and cerebellar lesions). On the basis of one of the largest studies of precision grip control after stroke, the excessive GF was attributed to impaired sensory feedback (Blennerhassett et al., 2007). The lack of temporal coupling was linked to deficits in developing internal models. Temporal coupling impairments could also be due to disturbed feedback transmission – this implies that an inability to update the sensorimotor memory. Predictive and reactive nature of deficits in precision grip have also been assessed further using the self or examiner brisk loading bimanual system described for children with HCP in order to test both of them separately. Deficits highlighted in the paretic hand were similar to those observed on the more affected hand of children with HCP. In predictive conditions, while an anticipatory control was present, temporal deficits were shown after brisk load increase. In reactive conditions, a slight increase was observed in the reflex latency. The less-affected hand did not differ from age- and sex-matched healthy controls (Dispa et al., 2014).

Previous studies including analysis of precision grip in the less-affected hand in patients with unilateral middle cerebral artery stroke (Quaney et al., 2005) showed an increase GF at lift-off across dynamic and static portions of the grip–lift task. In patients with subcortical lesion (subacute phase), the ipsilesional hand was also affected with a slowing of movements and poor coordination of grip and lift forces (Nowak et al., 2007).

Both in stroke patients and children with HCP, major precision grip impairments are observed in the paretic hand. Many of the deficits highlighted in the prehension of children with HCP and stroke patients present similarities: excessive GF, lack of GF and LF coupling, negative LF at the onset of the movement, release impairments, and a decreased ability to adapt to different loads (Table 1). These impairments are likely due to impairments in both predictive and reactive process.

## Implications for Rehabilitation

Various implications for rehabilitation could arise from precision grip deficits described in both congenital and acquired hemiparesis. First, as a consequence of deficits observed in the integration of sensory feedback in the anticipatory control of precision grip, one could suggest training of sensory abilities, as these are needed for precision grip tasks and are traditionally considered as a prerequisite for the performance of prehension tasks (Moberg, 1958; Jones, 1996; Gordon et al., 1999). The idea that recovery of the sensory system is needed and/or useful for motor recovery is widespread (Peurala et al., 2002; Smania et al., 2003; Blennerhassett et al., 2007; Conforto et al., 2007; Shirahashi et al., 2007), and partly forms the basis of some treatment approaches such as neurodevelopmental treatment (NDT). A recent paper on tactile spatial resolution in acute stroke patients even suggests that the link between sensory and motor modalities is such that pure motor stroke patients temporarily develop a hypersensibility on the paretic hand to compensate for the absence of movement (Doh et al., 2008). This implies a strong link between sensory and motor functions. Therefore, one could focus rehabilitation mainly on sensory modalities with the expectation of enhancing motor function (Bumin and Kayihan, 2001). This could be especially true at the beginning of rehabilitation where the motor deficit is greatest, and there is typically a lack of active movement. However, this strong link between sensory and motor function can be questioned as a result of the lack of correlation between sensory dysfunction and motor deficits (Fugl-Meyer et al., 1975; Bleyenheuft and Thonnard, 2011). The correlation between sensory modalities and independence in everyday life activities has been described only as weak to moderate in stroke patients (Carey and Matyas, 2011). Still in stroke patients, a recent single case report shows no effect of a change in proprioceptive abilities on motor recovery (Helliwell, 2009). Regarding children with HCP, Cooper et al. (1995) stated that “The extent of sensory loss did not mirror the severity of motor deficit.” Moreover, a study that included a large sample of children with cerebral palsy demonstrated that neither tactile pressure detection nor proprioception was related to manual ability (Arnould et al., 2007).

Many attempts have been made to increase sensory performance of stroke patients through long-term rehabilitation (Dannenbaum and Dykes, 1988; Carey et al., 1993; Yekutiel and Guttman, 1993; Carey and Matyas, 2005; Voller et al., 2006; Sullivan and Hedman, 2007), or transiently via local anesthesia to the intact hand (Doh et al., 2008) or transcranial direct current stimulation (Ragert et al., 2008). A recent review suggested that while there is a larger number of studies describing efficacy of passive sensory training than active sensory training (Schabrun and Hillier, 2009), there is “insufficient evidence to support or refute their effectiveness in improving sensory impairment, upper-limb function” (Doyle et al., 2010). However, a critical appraisal for this review added some relevant approaches to “active” sensory retraining (Carey et al., 2010) and a recent study showed the effectiveness of neurorehabilitation on sensation (Carey et al., 2011). Sensory cueing has also been recently described as useful for improving voluntary arm use (Fong et al., 2011).

Motor recovery is linked to cortical reorganization (Rehme et al., 2011a,b). Many strategies have also been developed to increase the motor performance of precision grip in stroke patients: Quaney et al. (2010) have recently shown interesting results on visuomotor training and suggested also to use retraining of the ipsilesional hand to improve function of contralesional hand. Strength training in the paretic hand improves both strength and function (Harris and Eng, 2010). This type of treatment based on force training is congruent with studies reporting that grip strength of the paretic hand best predicts upper-limb performance (Boissy et al., 1999; Harris and Eng, 2007). Recently, transcranial direct continuous stimulation (dual-tDCS) applied simultaneously over the ipsilesional (anodal) and contralateral (cathodal) primary motor cortices for a short duration (20 min) has been demonstrated as transiently improving precision grip in patients with stroke (Lefebvre et al., 2014). Functional electrical stimulation therapy is described as efficacious for rehabilitation of reach and grasp function (Thrasher et al., 2008) Constraint-induced movement therapy (CIMT) is also designed to increase hand motor function (see Eliasson and Gordon, 2008; Sawaki et al., 2008; Gordon and Friel, 2009; Sakzewski et al., 2009). Inhibitory repetitive transcranial magnetic stimulation (rTMS – 1 Hz) over the unaffected primary motor cortex improves the timing of grasping and lifting with the affected hand (Dafotakis et al., 2008). Mirror therapy (Michielsen et al., 2011) and mental training have a positive effect on the hand function (Müller et al., 2007; Nilsen et al., 2010; Prasad et al., 2010; Ietswaart et al., 2011). Robot-assisted therapies also have potential for prehension recovery (Ziherl et al., 2010; Merians et al., 2011). The monitoring of recovery and treatment strategies after stroke through motion analysis has been reviewed (Nowak, 2008). The nature of hand motor impairment and its treatment after stroke has also been reviewed (Raghavan, 2007), as well as treatment’s intensity (Cooke et al., 2010). Whatever the strategy used to re-train precision grip, a large cohort study shows that the likelihood to regain some dexterity is strongly linked to the presence of early voluntary extension of the fingers and abduction of the shoulder (Nijland et al., 2010; Kong et al., 2011).

In children with HCP, many types of training have provided evidence of increasing precision grip performance: short term training effects were reported (Wenzelburger et al., 2005), training based on visual feedback (Valvano and Newell, 1998), and intensive training with CIMT (Charles et al., 2001).

In stroke patients, like in children with HCP, the ipsilesional hand has only subtle deficits (Gordon et al., 1999; Quaney et al., 2005; Nowak et al., 2007). *Therefore, the performance of this less-affected hand is of interest for rehabilitation purposes*. As the patients are able to correctly program precision tasks with the non-involved hand, this ability could be used either to transfer intact sensory information from the non-involved to the involved hand, or to construct a template for the appropriate motor command. The use of both hands to establish appropriate motor commands bilaterally has been developed with success in neurorehabilitation both in children with HCP (Hung et al., 2004; Gordon et al., 2007, 2008) and acquired hemiparesis (Brogårdh and Lexell, 2010; Hayner et al., 2010). In contrast, while the transfer of information from the non-involved to the involved hand has been clearly demonstrated (Raghavan et al., 2006; Steenbergen et al., 2008), likely involving the corpus callosum, neurorehabilitation based on the alternate use of both hands (kinematic mirroring), starting with the non-paretic hand has not yet been tested. While a new neurorehabilitation strategy based on this principle could be interesting if it enables acquisition of a valid motor plan that could be transferred from the non-paretic to the paretic hand, the effectiveness of this scheme could be questioned because of inter-hemispheric influences (Rouiller et al., 1994; Di Lazzaro et al., 1999; Mochizuki et al., 2004; Westlake and Nagarajan, 2011). Through transcallosal interactions, each primary motor cortex when stimulated has an inhibitory action on the opposite motor cortex. Future studies based on kinematic mirroring should provide interesting clues on the possibility of transfer from the less-affected to the paretic hand (Ward and Cohen, 2004; Duque et al., 2005).

## Limitations

The discussion of similarities in hand impairments of children with HCP and adult acquired lesion would have benefited from investigations both in adults with HCP and in children with later acquired lesion (3 years and older). While several studies are available for adults with HCP, especially in the field of functioning and quality of life (Roebroeck et al., 2009; Mesterman et al., 2010), none specifically focus on their hand function and precision grip. However, the stability of gross motor function classification (GMFCS) after the age of 12 years (McCormick et al., 2007; Hanna et al., 2009) and the absence of decrease in functional performance with age in longitudinal studies (Eliasson et al., 2006; Blank and Kluger, 2009) suggest that the deficit relative to healthy peers probably remains stable when children with HCP become adults. For children with later acquired brain injury, no information is available on hand function. Therefore, it would be interesting in future studies to specifically focus on hand function in children with acquired brain injury after the age of 3 years as well as in adults with HCP.

## Conclusion

In this review, we have documented impairments in predictive motor control of patients with congenital and acquired hemiparesis. The ability to use such information in an anticipatory manner (i.e., based on memory from prior manipulations) to scale their forces was present in both children and adults with hemiparesis. Patients with acquired or congenital hemiparesis had the ability – often with many trials for new objects/conditions – to predicatively scale their forces to different weights or surface friction. This highlights their remaining possibility to use predictive control, even if it is impaired in the paretic hand. In both congenital and acquired hemiparesis, it is suggested that the ipsilesional hand may aid the subsequent control of the contralesional hand. Indeed, even if it has subtle deficits, this hand has proven in both groups of patients an intact ability to aid in anticipatory control.

We initially aimed to determine whether the neonatal or adult occurrence of the lesion influences the type and importance of hand deficits. We hypothesized that the comparison of hand deficits in early lesions in children and acquired lesion in adults should help to direct neurorehabilitation of hand deficits to each pathology more specifically. Surprisingly, except for the more focused studies on different locations of stroke in adults (cerebellar, capsular stroke, etc.), we observed very few differences between the results of studies in children with HCP and stroke patients. The motor control deficits measured with precision grip were similar. Therefore, we suggest that while the therapeutic approach should be adapted to the age and the level of understanding of each patient, the underlying strategies of neurorehabilitation could be similar in adult stroke patients and children with HCP.

## Conflict of Interest Statement

The authors declare that the research was conducted in the absence of any commercial or financial relationships that could be construed as a potential conflict of interest.
